# Supplementary material for: The mosquito vectors that sustained malaria transmission during the Magude project despite the combined deployment of indoor residual spraying, insecticide-treated nets and mass-drug administration
Source: PLoS One. 2022 Sep 9;17(9):e0271427. doi: 10.1371/journal.pone.0271427 (PMC9462736; doi:10.1371/journal.pone.0271427)
Supplement: S1 File — (DOCX) [file pone.0271427.s001.docx]

**S1 Box: Exclusions criteria for mosquito collections**

| **Mosquito collections meeting the following criteria were excluded from the analyses.**   - The fan and/or light of the trap was not working; - Collection bottle was not properly attached; - Synthetic lure and/or artificial CO2 source not placed/connected properly. - The household resident(s) did not sleep under their LLIN next to the trap, - More than two people slept next to the trap; - Ants found in a collection bottle; - The bottle rotator had not completed all programmed rotations; - Collections started before 4pm or extended beyond 8am. |
| --- |
