# Supplementary material for: The mosquito vectors that sustained malaria transmission during the Magude project despite the combined deployment of indoor residual spraying, insecticide-treated nets and mass-drug administration
Source: PLoS One. 2022 Sep 9;17(9):e0271427. doi: 10.1371/journal.pone.0271427 (PMC9462736; doi:10.1371/journal.pone.0271427)

**S2 Sinusoidal function used to simulate ITN use based on observed values of ITN use**

$$f\left( x \right)=A\sin B\left( x-C \right)+D$$

Where x is the month, A is the amplitude of the variation which we modeled as $amplitude=\frac{{max(ITN}_{observed use})-min({ITN}_{observed use})}{2}$, B is the period, which for months is $\frac{2\pi}{12}$, C was adjusted for the sinusoidal function to follow the seasonality of ITN use and D is the minimum observed use (39.1%) plus the amplitude of the variation.


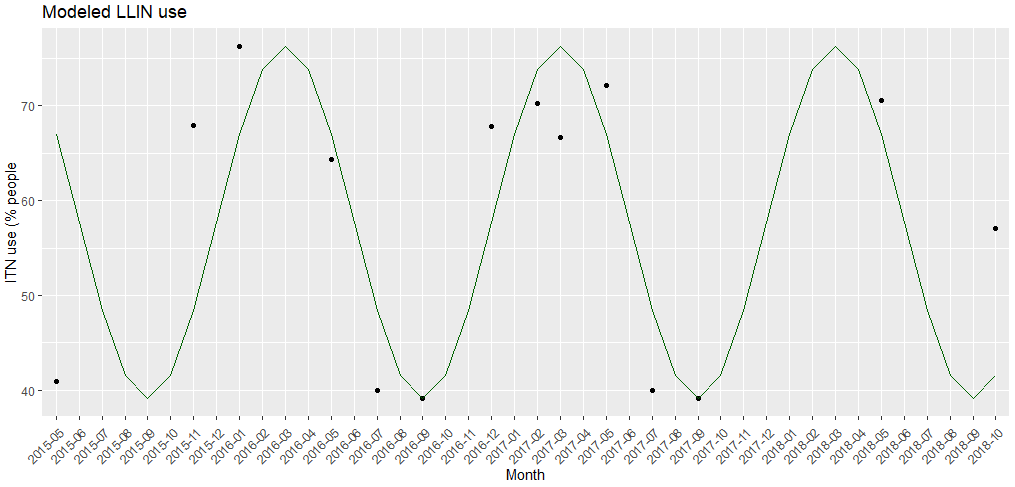

Supplement: S2 File — (DOCX) [file pone.0271427.s002.docx]
