# Supplementary material for: The mosquito vectors that sustained malaria transmission during the Magude project despite the combined deployment of indoor residual spraying, insecticide-treated nets and mass-drug administration
Source: PLoS One. 2022 Sep 9;17(9):e0271427. doi: 10.1371/journal.pone.0271427 (PMC9462736; doi:10.1371/journal.pone.0271427)
Supplement: S3 File — (DOCX) [file pone.0271427.s003.docx]

**S3 Detailed Sporozoite detection results**

**Table 1: Sporozoite rates per species during the entire study period (before and after intervention implementation)**

| **Vector species** | **Sporozoite Negative**  n (%) | **Sporozoite**  **Positive**  n (%) | **Not tested**  n (%) |
| --- | --- | --- | --- |
| An. arabiensis | 3020 (95.5%) | 32 (1%) | 111 (3.5%) |
| An. quadriannulatus | 59 (93.7%) | 0 (0%) | 4 (6.3%) |
| An. merus | 127 (94.1%) | 1 (0.7%) | 7 (5.2%) |
| An. gambiae.ss | 3 (100%) | 0 (0%) | 0 (0%) |
| Unidentified An..gambiae.s.l. | 61 (91%) | 1 (1.5%) | 5 (7.5%) |
| An. funestus.ss | 100 (79.4%) | 1 (0.8%) | 25 (19.8%) |
| An. parensis | 110 (60.1%) | 1 (0.5%) | 72 (39.3%) |
| An. leesoni | 2 (50%) | 0 (0%) | 2 (50%) |
| An. rivulorum | 15 (83.3%) | 0 (0%) | 3 (16.7%) |
| Unidentified An..funestus.s.l. | 34 (69.4%) | 0 (0%) | 15 (30.6%) |
| An. coustani | 6 (50%) | 0 (0%) | 6 (50%) |
| An. demeilloni | 1 (50%) | 0 (0%) | 1 (50%) |
| An. garnhami | 1 (100%) | 0 (0%) | 0 (0%) |
| An. listeri | 0 (0%) | 0 (0%) | 10 (100%) |
| An. marshallii | 3 (100%) | 0 (0%) | 0 (0%) |
| An. multicolor | 0 (0%) | 0 (0%) | 1 (100%) |
| An. pharoensis | 46 (46.5%) | 0 (0%) | 53 (53.5%) |
| An. pretoriensis | 1 (100%) | 0 (0%) | 0 (0%) |
| An. rufipes | 8 (9.5%) | 0 (0%) | 76 (90.5%) |
| An. salbaii | 0 (0%) | 0 (0%) | 1 (100%) |
| An. squamosus | 16 (4.1%) | 1 (0.3%) | 372 (95.6%) |
| An. tenebrosus | 1 (11.1%) | 0 (0%) | 8 (88.9%) |
| An. ziemani | 5 (4.3%) | 0 (0%) | 111 (95.7%) |

**Fig 1 Monthly proportion of collected mosquitoes that underwent sporozoite detection**


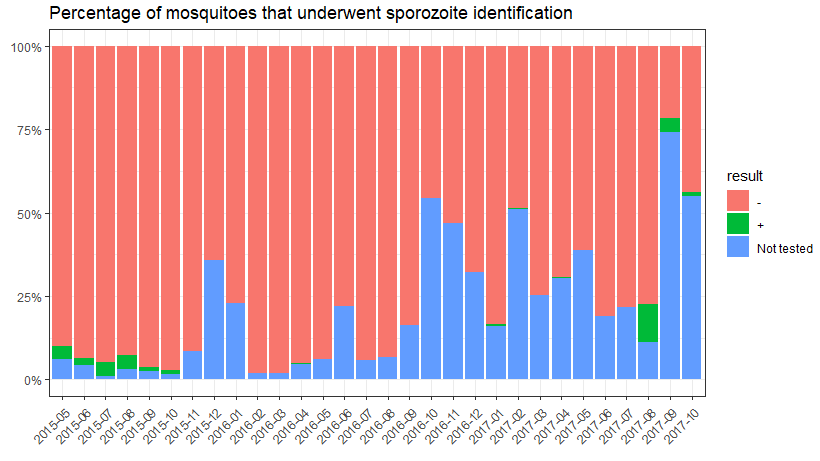


**Table 2: Monthly percentage of sporozoite positive *Anopheles* mosquitos**

| **Month** | **Number sporozoite positive** | **Number tested** | **Positivity rate (%)** |
| --- | --- | --- | --- |
| May-15 | 171 | 7 | 4.09 |
| Jun-15 | 91 | 2 | 2.2 |
| Jul-15 | 118 | 5 | 4.24 |
| Aug-15 | 94 | 4 | 4.26 |
| Sep-15 | 78 | 1 | 1.28 |
| Oct-15 | 68 | 1 | 1.47 |
| Nov-15 | 32 | 0 | 0 |
| Dec-15 | 9 | 0 | 0 |
| Jan-16 | 44 | 0 | 0 |
| Feb-16 | 101 | 0 | 0 |
| Mar-16 | 335 | 0 | 0 |
| Apr-16 | 657 | 2 | 0.3 |
| May-16 | 78 | 0 | 0 |
| Jun-16 | 64 | 0 | 0 |
| Jul-16 | 133 | 0 | 0 |
| Aug-16 | 28 | 0 | 0 |
| Sep-16 | 26 | 0 | 0 |
| Oct-16 | 37 | 0 | 0 |
| Nov-16 | 57 | 0 | 0 |
| Dec-16 | 40 | 0 | 0 |
| Jan-17 | 111 | 1 | 0.9 |
| Feb-17 | 236 | 1 | 0.42 |
| Mar-17 | 370 | 0 | 0 |
| Apr-17 | 369 | 1 | 0.27 |
| May-17 | 101 | 0 | 0 |
| Jun-17 | 72 | 0 | 0 |
| Jul-17 | 18 | 0 | 0 |
| Aug-17 | 55 | 7 | 12.73 |
| Sep-17 | 26 | 4 | 15.38 |
| Oct-17 | 37 | 1 | 2.7 |

**Table 3: List of sporozoite positive *Anopheles* mosquitos**

| Date of mosquito collection | ELISA result | Species | Sentinel site | Trap location |
| --- | --- | --- | --- | --- |
| 2015-05-26 | + | An. arabiensis | Mapulanguene | indoor |
| 2015-05-21 | + | An. arabiensis | Muginge | indoor |
| 2015-05-21 | + | An. parensis | Muginge | indoor |
| 2015-05-19 | + | An. arabiensis | Panjane | indoor |
| 2015-05-06 | + | An. arabiensis | Chicutso | indoor |
| 2015-05-28 | + | An. arabiensis | Chicutso | indoor |
| 2015-05-28 | + | An. arabiensis | Chicutso | indoor |
| 2015-06-10 | + | An. arabiensis | Motaze | indoor |
| 2015-06-19 | + | An. funestus ss | Muginge | indoor |
| 2015-08-07 | + | An. arabiensis | Magude Sede | indoor |
| 2015-07-23 | + | An. arabiensis | Muginge | indoor |
| 2015-07-23 | + | An. arabiensis | Muginge | indoor |
| 2015-07-23 | + | An. arabiensis | Muginge | indoor |
| 2015-07-23 | + | An. arabiensis | Muginge | indoor |
| 2015-07-30 | + | An. arabiensis | Chicutso | indoor |
| 2015-08-27 | + | An. arabiensis | Chicutso | indoor |
| 2015-08-27 | + | An. arabiensis | Chicutso | indoor |
| 2015-08-27 | + | An. arabiensis | Chicutso | indoor |
| 2015-09-10 | + | An. arabiensis | Chicutso | indoor |
| 2015-10-16 | + | An. squamosus | Motaze | indoor |
| 2016-04-28 | + | An. arabiensis | Muginge | indoor |
| 2016-04-29 | + | An. arabiensis | Muginge | indoor |
| 2017-01-27 | + | An. arabiensis | Muginge | indoor |
| 2017-02-24 | + | An. arabiensis | Muginge | indoor |
| 2017-04-28 | + | An. arabiensis | Muginge | indoor |
| 2017-08-01 | + | An. gambiae s.l. | Chicutso | outdoor |
| 2017-08-02 | + | An. arabiensis | Chicutso | indoor |
| 2017-08-02 | + | An. arabiensis | Chicutso | indoor |
| 2017-08-11 | + | An. arabiensis | Magude Sede | indoor |
| 2017-09-13 | + | An. arabiensis | Muginge | indoor |
| 2017-09-13 | + | An. merus | Muginge | indoor |
| 2017-08-15 | + | An. arabiensis | Muginge | indoor |
| 2017-08-15 | + | An. arabiensis | Muginge | indoor |
| 2017-08-15 | + | An. arabiensis | Muginge | indoor |
| 2017-09-22 | + | An. arabiensis | Magude Sede | indoor |
| 2017-09-21 | + | An. arabiensis | Magude Sede | indoor |
| 2017-10-24 | + | An. arabiensis | Muginge | outdoor |
