# Supplementary material for: The mosquito vectors that sustained malaria transmission during the Magude project despite the combined deployment of indoor residual spraying, insecticide-treated nets and mass-drug administration
Source: PLoS One. 2022 Sep 9;17(9):e0271427. doi: 10.1371/journal.pone.0271427 (PMC9462736; doi:10.1371/journal.pone.0271427)

**S4 Detailed model results**

1. **Best model: With covariates lagged one month and considering only the intervention period**


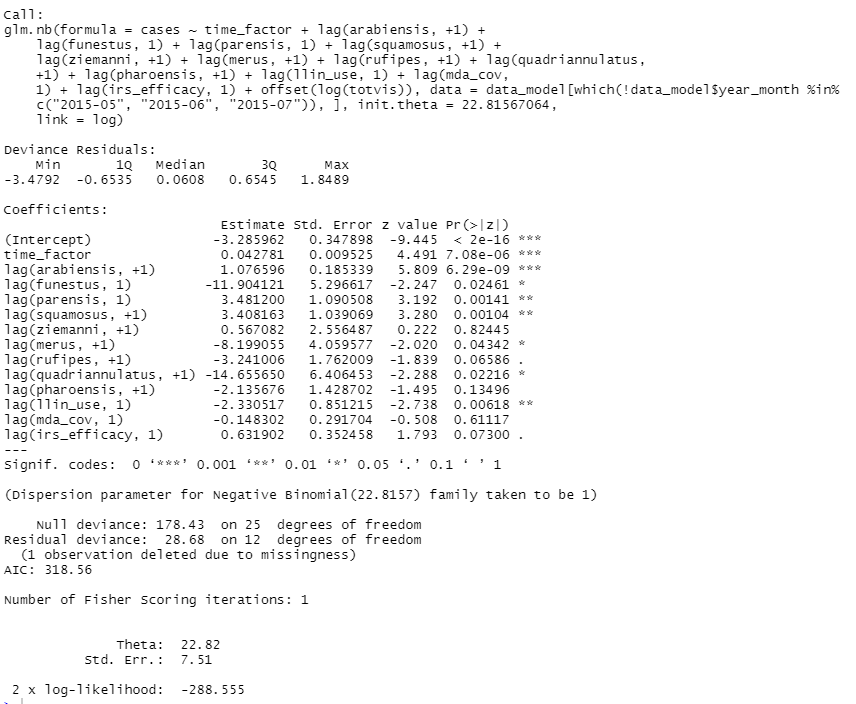


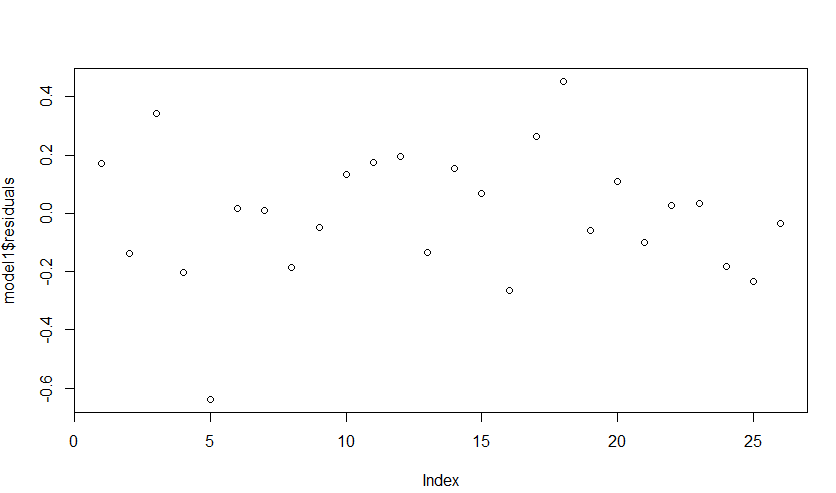


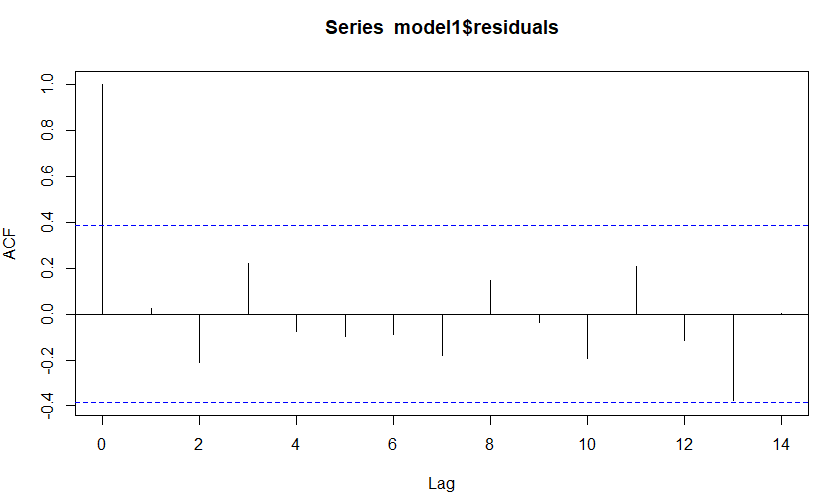


1. **Model 2: With unlagged covariates and considering only the intervention period**


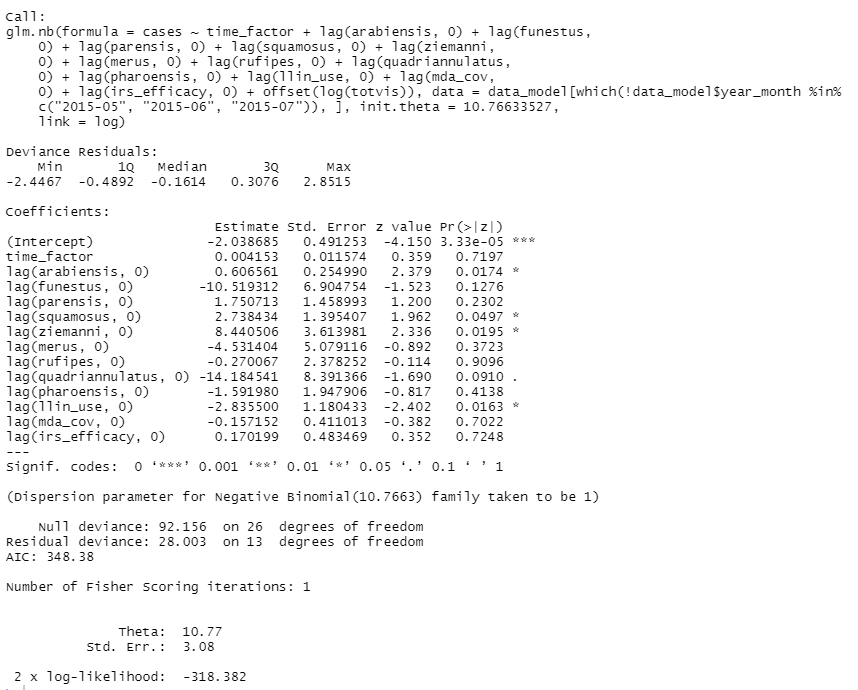


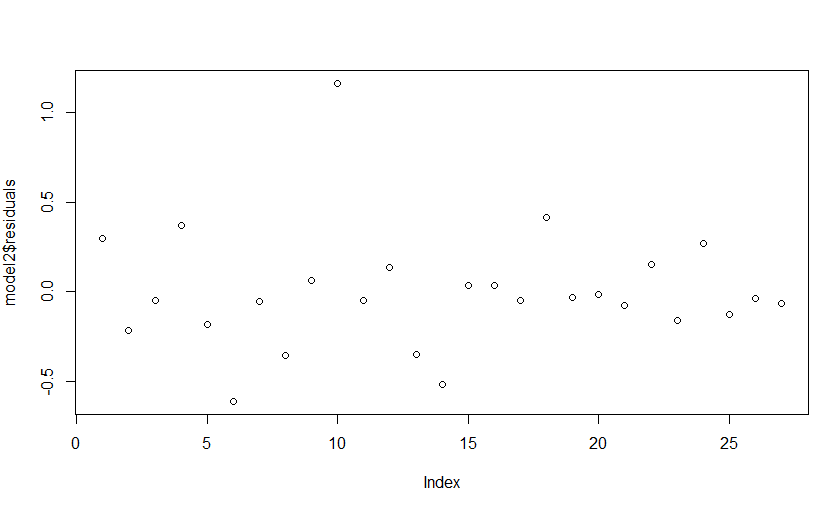


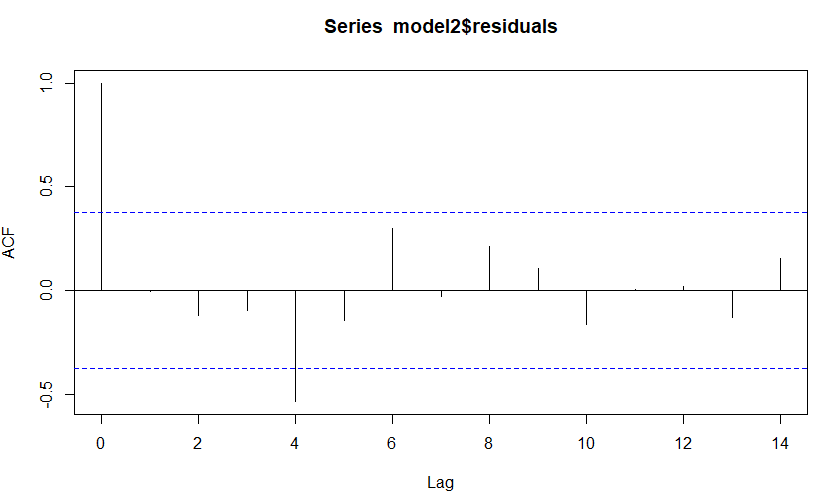


1. **Model 3: With covariates lagged one month and considering the intervention and baseline period**


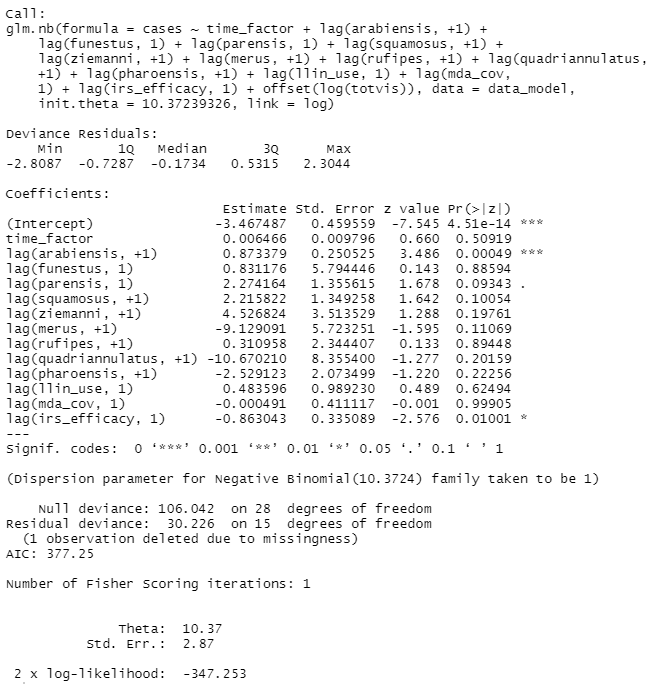


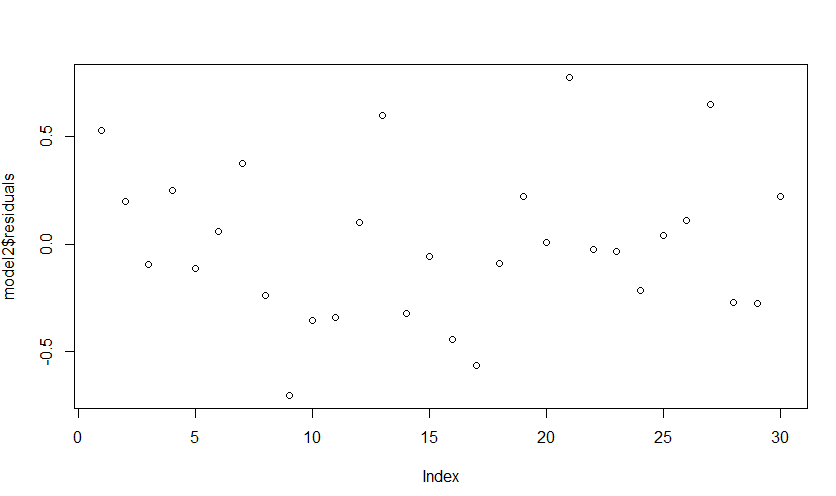


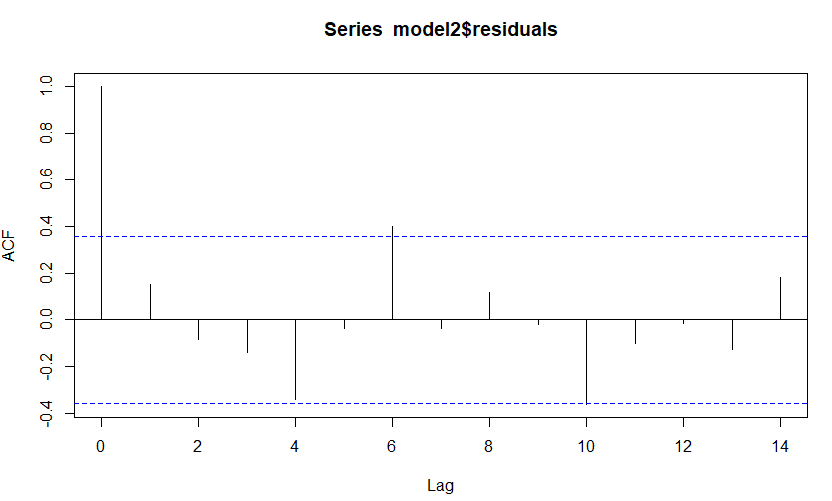


1. **Model 4: With unlagged covariates and considering the intervention and baseline period**


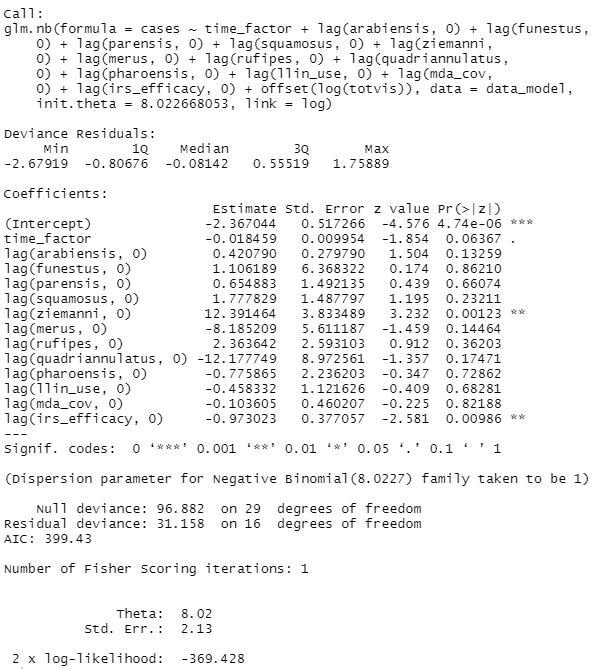


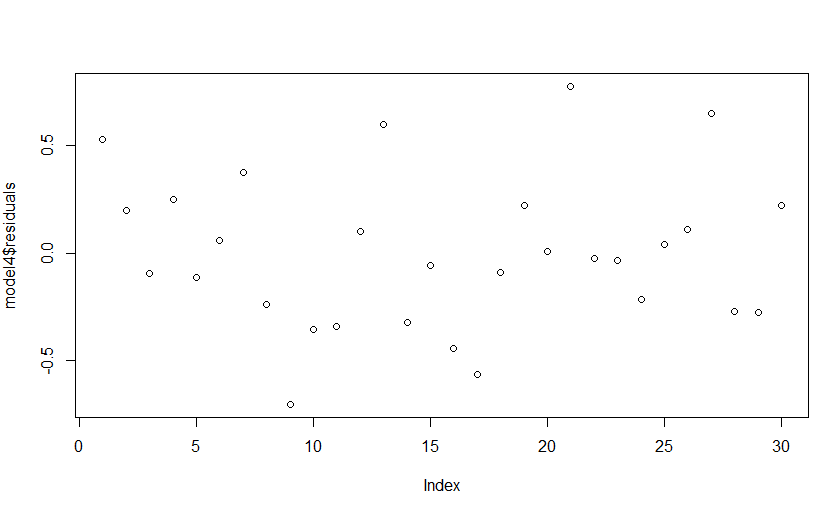


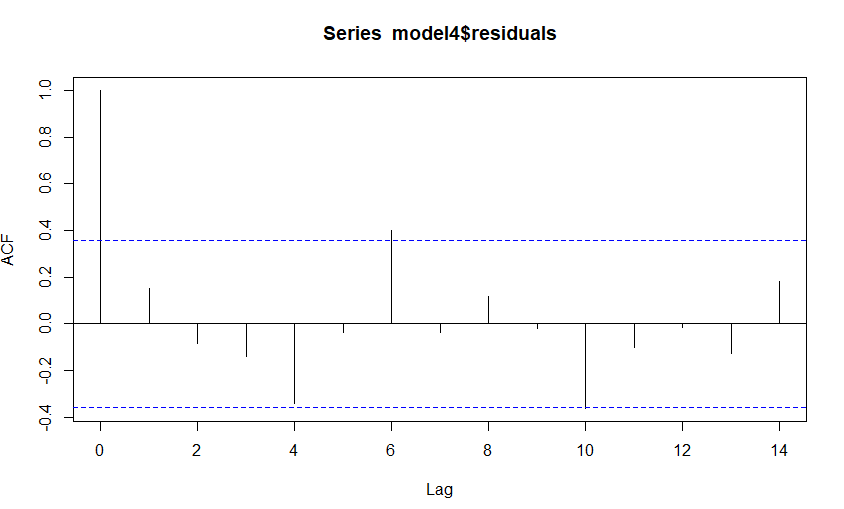

Supplement: S4 File — (DOCX) [file pone.0271427.s004.docx]
